# Supplementary material for: Temporal aspects of unrealistic optimism and robustness of this bias: A longitudinal study in the context of the COVID-19 pandemic
Source: PLoS One. 2022 Dec 15;17(12):e0278045. doi: 10.1371/journal.pone.0278045 (PMC9754208; doi:10.1371/journal.pone.0278045)
Supplement: S1 Table — (DOCX) [file pone.0278045.s001.docx]

| **Wave** | **CO_index_ categories** | **Frequency** | **Percent** | **Cumulative Percent** |
| --- | --- | --- | --- | --- |
| **1** | CO_index_ < 0 (pessimism) | 13 | 10.83 | 10.83 |
|  | CO_index_ = 0 (unbiased) | 94 | 78.33 | 89.17 |
|  | CO_index_ > 0 (optimism) | 13 | 10.83 | 100 |
|  | Missing | 0 | 0 |  |
|  | Total | 120 | 100 |  |
| **2** | CO_index_ < 0 (pessimism) | 11 | 9.17 | 9.17 |
|  | CO_index_ = 0 (unbiased) | 68 | 56.67 | 65.83 |
|  | CO_index_ > 0 (optimism) | 41 | 34.17 | 100 |
|  | Missing | 0 | 0 |  |
|  | Total | 120 | 100 |  |
| **3** | CO_index_ < 0 (pessimism) | 10 | 8.33 | 8.33 |
|  | CO_index_ = 0 (unbiased) | 70 | 58.33 | 66.67 |
|  | CO_index_ > 0 (optimism) | 40 | 33.33 | 100 |
|  | Missing | 0 | 0 |  |
|  | Total | 120 | 100 |  |
| **4** | CO_index_ < 0 (pessimism) | 26 | 21.67 | 21.67 |
|  | CO_index_ = 0 (unbiased) | 70 | 58.33 | 80 |
|  | CO_index_ > 0 (optimism) | 24 | 20 | 100 |
|  | Missing | 0 | 0 |  |
|  | Total | 120 | 100 |  |
| **5** | CO_index_ < 0 (pessimism) | 25 | 20.83 | 20.83 |
|  | CO_index_ = 0 (unbiased) | 72 | 60 | 80.83 |
|  | CO_index_ > 0 (optimism) | 23 | 19.17 | 100 |
|  | Missing | 0 | 0 |  |
|  | Total | 120 | 100 |  |
| **6** | CO_index_ < 0 (pessimism) | 19 | 15.83 | 15.83 |
|  | CO_index_ = 0 (unbiased) | 66 | 55 | 70.83 |
|  | CO_index_ > 0 (optimism) | 35 | 29.17 | 100 |
|  | Missing | 0 | 0 |  |
|  | Total | 120 | 100 |  |
| **7** | CO_index_ < 0 (pessimism) | 14 | 11.67 | 11.67 |
|  | CO_index_ = 0 (unbiased) | 64 | 53.33 | 65 |
|  | CO_index_ > 0 (optimism) | 42 | 35 | 100 |
|  | Missing | 0 | 0 |  |
|  | Total | 120 | 100 |  |
| **8** | CO_index_ < 0 (pessimism) | 21 | 17.5 | 17.5 |
|  | CO_index_ = 0 (unbiased) | 49 | 40.83 | 58.33 |
|  | CO_index_ > 0 (optimism) | 50 | 41.67 | 100 |
|  | Missing | 0 | 0 |  |
|  | Total | 120 | 100 |  |
| **9** | CO_index_ < 0 (pessimism) | 12 | 10 | 10 |
|  | CO_index_ = 0 (unbiased) | 48 | 40 | 50 |
|  | CO_index_ > 0 (optimism) | 60 | 50 | 100 |
|  | Missing | 0 | 0 |  |
|  | Total | 120 | 100 |  |
| **10** | CO_index_ < 0 (pessimism) | 14 | 11.67 | 11.67 |
|  | CO_index_ = 0 (unbiased) | 46 | 38.33 | 50 |
|  | CO_index_ > 0 (optimism) | 60 | 50 | 100 |
|  | Missing | 0 | 0 |  |
|  | Total | 120 | 100 |  |
| **11** | CO_index_ < 0 (pessimism) | 14 | 11.67 | 11.67 |
|  | CO_index_ = 0 (unbiased) | 48 | 40 | 51.67 |
|  | CO_index_ > 0 (optimism) | 58 | 48.33 | 100 |
|  | Missing | 0 | 0 |  |
|  | Total | 120 | 100 |  |
| **12** | CO_index_ < 0 (pessimism) | 20 | 16.67 | 16.67 |
|  | CO_index_ = 0 (unbiased) | 53 | 44.17 | 60.83 |
|  | CO_index_ > 0 (optimism) | 47 | 39.17 | 100 |
|  | Missing | 0 | 0 |  |
|  | Total | 120 | 100 |  |
| **13** | CO_index_ < 0 (pessimism) | 21 | 17.5 | 17.5 |
|  | CO_index_ = 0 (unbiased) | 54 | 45 | 62.5 |
|  | CO_index_ > 0 (optimism) | 45 | 37.5 | 100 |
|  | Missing | 0 | 0 |  |
|  | Total | 120 | 100 |  |
| **14** | CO_index_ < 0 (pessimism) | 13 | 10.83 | 10.83 |
|  | CO_index_ = 0 (unbiased) | 58 | 48.33 | 59.17 |
|  | CO_index_ > 0 (optimism) | 49 | 40.83 | 100 |
|  | Missing | 0 | 0 |  |
|  | Total | 120 | 100 |  |
| **15** | CO_index_ < 0 (pessimism) | 10 | 8.33 | 8.33 |
|  | CO_index_ = 0 (unbiased) | 58 | 48.33 | 56.67 |
|  | CO_index_ > 0 (optimism) | 52 | 43.33 | 100 |
|  | Missing | 0 | 0 |  |
|  | Total | 120 | 100 |  |
| **16** | CO_index_ < 0 (pessimism) | 8 | 6.67 | 6.67 |
|  | CO_index_ = 0 (unbiased) | 51 | 42.5 | 49.17 |
|  | CO_index_ > 0 (optimism) | 61 | 50.83 | 100 |
|  | Missing | 0 | 0 |  |
|  | Total | 120 | 100 |  |
