# Supplementary material for: Temporal aspects of unrealistic optimism and robustness of this bias: A longitudinal study in the context of the COVID-19 pandemic
Source: PLoS One. 2022 Dec 15;17(12):e0278045. doi: 10.1371/journal.pone.0278045 (PMC9754208; doi:10.1371/journal.pone.0278045)
Supplement: S2 Table — (DOCX) [file pone.0278045.s002.docx]

| contrast | wave | η_p_² | SE | df | t.ratio | p.value |
| --- | --- | --- | --- | --- | --- | --- |
| Self - Others | 1 | -.200 | .116 | 119 | -1.728 | .087 |
| Self - Others | 2 | -.908 | .158 | 119 | -5.737 | .000 |
| Self - Others | 3 | -.958 | .174 | 119 | -5.492 | .000 |
| Self - Others | 4 | -.225 | .212 | 119 | -1.060 | .291 |
| Self - Others | 5 | -.492 | .266 | 119 | -1.845 | .068 |
| Self - Others | 6 | -1.092 | .260 | 119 | -4.202 | .000 |
| Self - Others | 7 | -1.850 | .307 | 119 | -6.032 | .000 |
| Self - Others | 8 | -1.925 | .357 | 119 | -5.386 | .000 |
| Self - Others | 9 | -2.825 | .345 | 119 | -8.194 | .000 |
| Self - Others | 10 | -2.500 | .325 | 119 | -7.688 | .000 |
| Self - Others | 11 | -1.950 | .297 | 119 | -6.555 | .000 |
| Self - Others | 12 | -.958 | .316 | 119 | -3.034 | .003 |
| Self - Others | 13 | -.933 | .283 | 119 | -3.298 | .001 |
| Self - Others | 14 | -1.492 | .285 | 119 | -5.238 | .000 |
| Self - Others | 15 | -1.825 | .280 | 119 | -6.524 | .000 |
| Self - Others | 16 | -2.392 | .287 | 119 | -8.346 | .000 |
