# Supplementary figures and images for: Temporal aspects of unrealistic optimism and robustness of this bias: A longitudinal study in the context of the COVID-19 pandemic
Source: PLoS One. 2022 Dec 15;17(12):e0278045. doi: 10.1371/journal.pone.0278045 (PMC9754208; doi:10.1371/journal.pone.0278045)

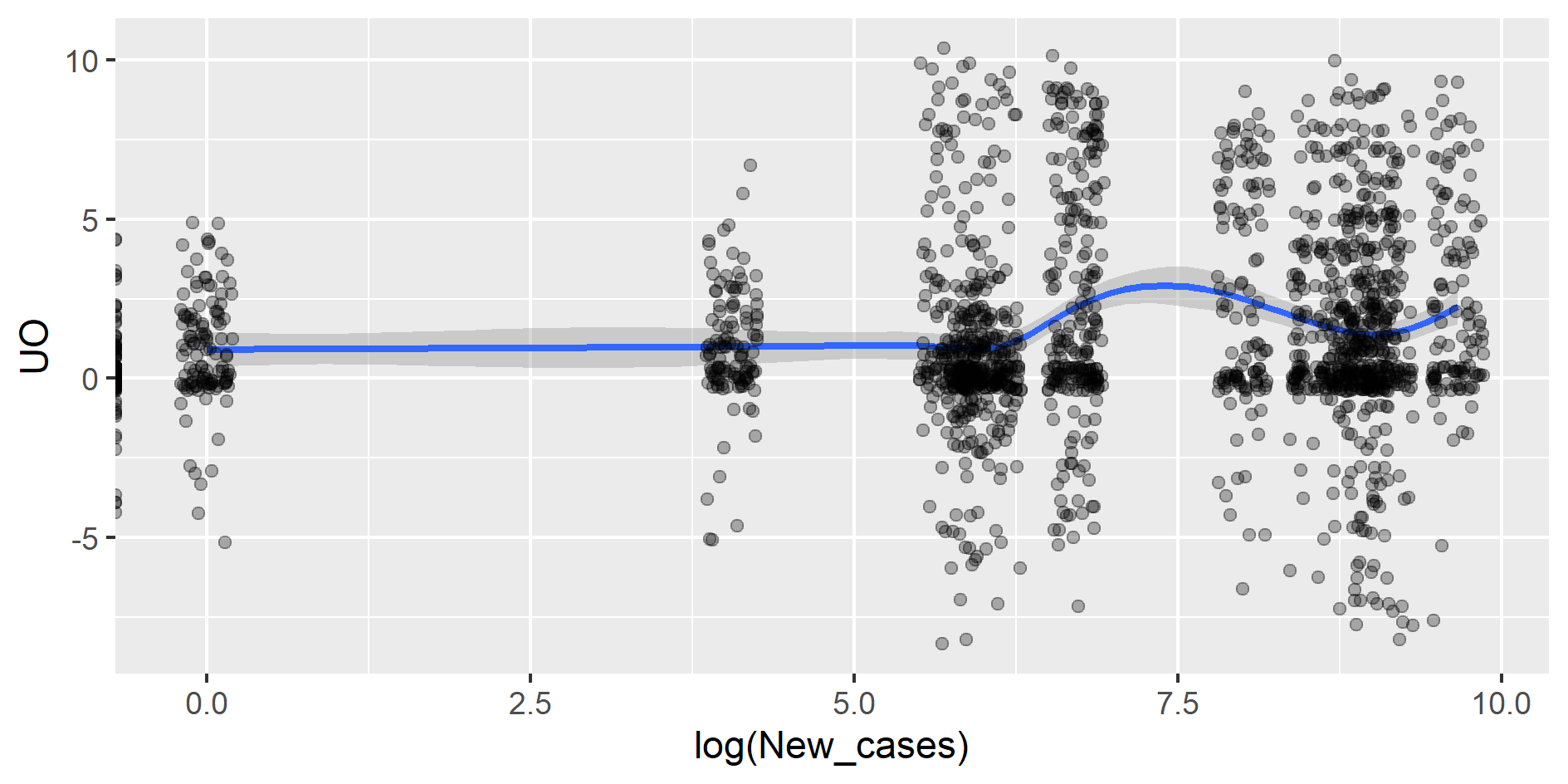

Supplement: S1 Fig — (TIF) [file pone.0278045.s003.tif]

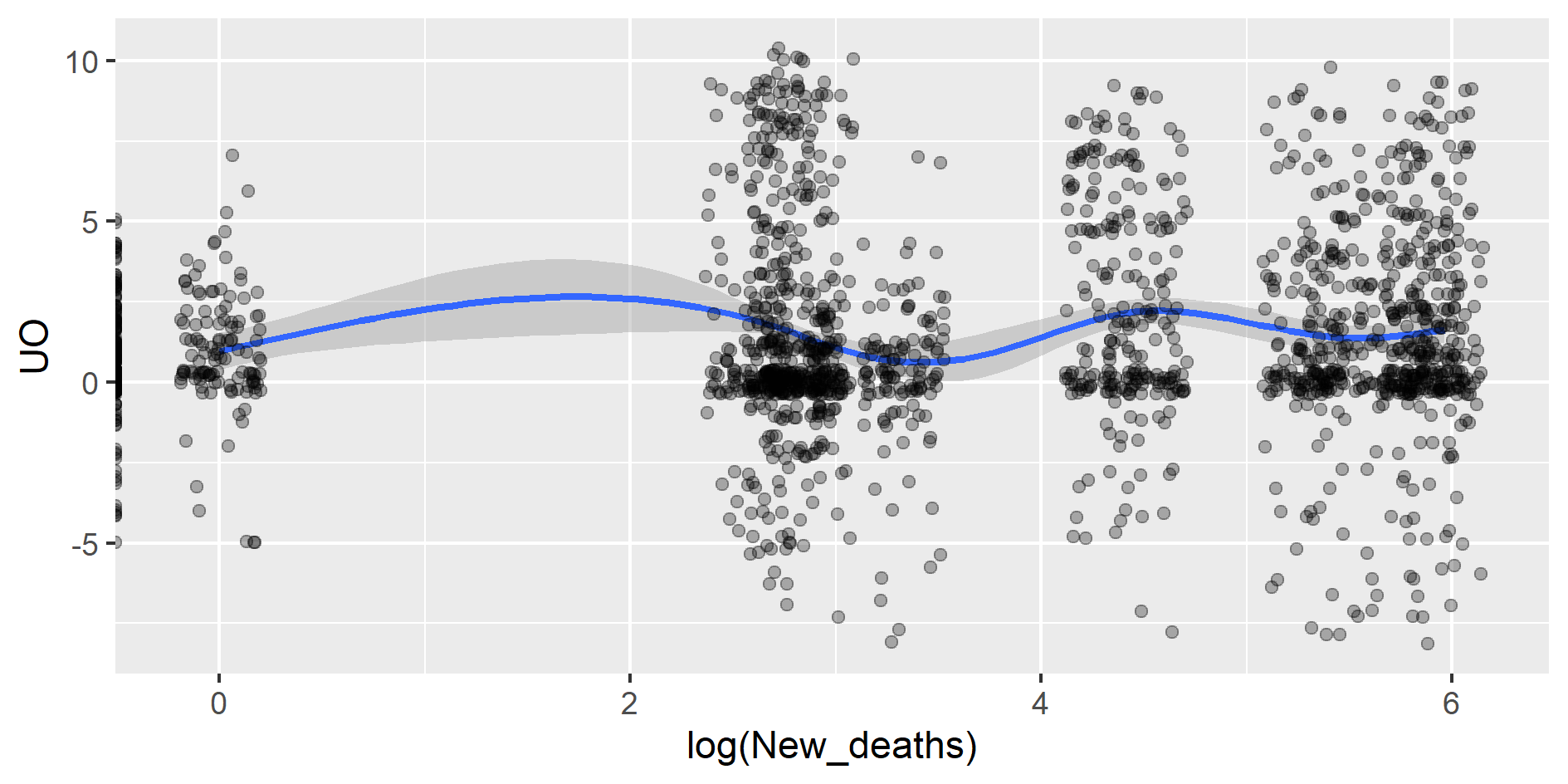

Supplement: S2 Fig — (TIF) [file pone.0278045.s004.tif]

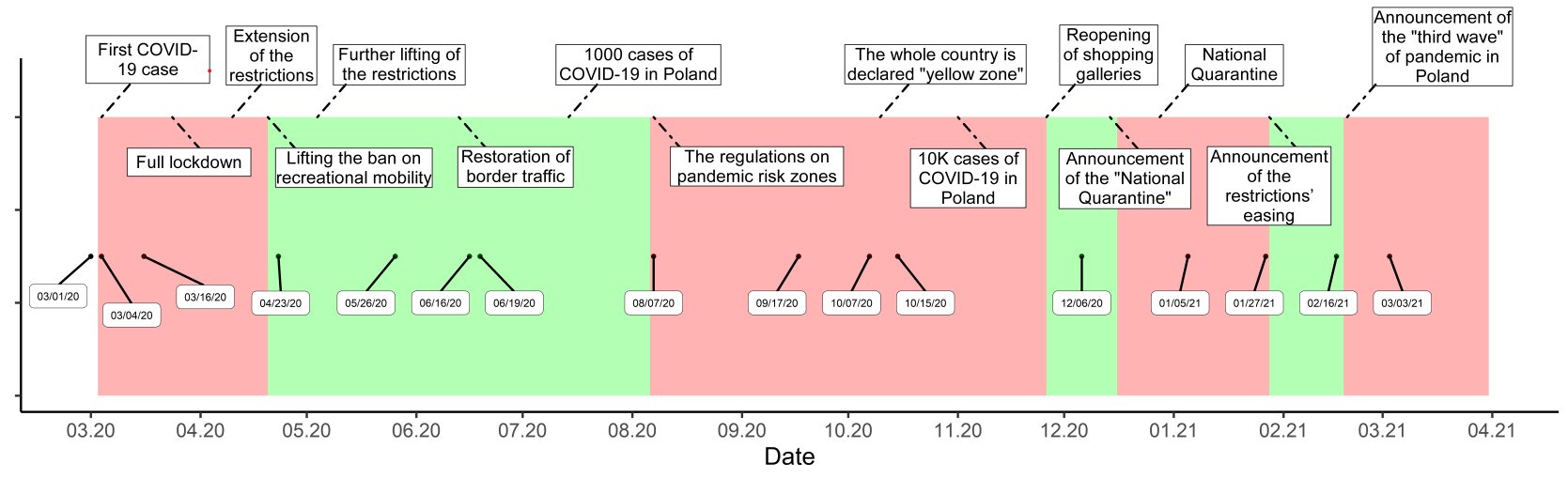

Supplement: S3 Fig — (PNG) [file pone.0278045.s005.png]
