# Supplementary material for: Temporal aspects of unrealistic optimism and robustness of this bias: A longitudinal study in the context of the COVID-19 pandemic
Source: PLoS One. 2022 Dec 15;17(12):e0278045. doi: 10.1371/journal.pone.0278045 (PMC9754208; doi:10.1371/journal.pone.0278045)
Supplement: Attachment — Submitted filename: PLOSONE_rev.docx [file pone.0278045.s008.docx]

Barbara Dolińska

Opole University

Temporal aspects of unrealistic optimism and robustness of this bias. A longitudinal study in teh context of the COVID-19 pandemic

Thank you for letting me read this paper and learn from the study described.

In this article the Authors present a very intriguing research concerning unrealistic optimism. For 12 months 120 participants were consequetivelly asked to estimate their own and their peers’ risk of COVID-19 infection. In this longitudinal study, the Authors were able to observe the robustness of the UO bias and to investigate the relations between the level of threat (objective information about the numer of cases and deaths as well as information about political decisions) and the magnitude of unrealistic optimism.

Overall, I found the paper very interesting to read. The manuscript is clearly written and the pattern of results obtained is intriguing. The theory and results obtained are relevant and contribute to the current body of knowledge, and this is why I think that after some (very minor) alternations are made to it, the article should be accepted.

(1) At the very begining of the article, the Authors introduce the term ”unrealistic optimism”. As authors most probably know, in psychology this term is used in two meanings:

- unrealistic absolute optimism

- unrealistic comparative optimism

(see: Shepperd, Klein, Waters & Weinstein, 2013 (Taking stock af unrealistic optimism – *Perspectives on Psychological Science*)

The authors decided to investigate comparative unrealistic optimism in their research (and without any doubts they were right, only such a measure is justified here). However, it is worth writing in the paper some words about the absolute optimism phenomenon (just 2 – 3 sentences). Thanks to this, the reader will have no doubts how the authors treated the unrealistic optimism and what they actually measured.

(2) I am very impressed by the authors longitudinal research on the dynamics of the unrealistic optimism. As far as I know, nobody in the world has investigated optimistic bias so many times in such a long period (one year!). Authors have shown changes in the level of unrealistic optimism under the influence of various factors.

The question came to my mind to what extend the level of unrealistic optimism is stable in normal (typical) situations. For ecample: does the level of unrealistic otpimism in relations to cancer or being the victim of car accident change over time? Of course, I am aware that the Authors do not know the answer to this question (I do not know it either), but perhaps it is worth asking such a rhetorical question in the section ”Limitation and directions of further research”.
